# Implementation of an infant male circumcision programme, Pakistan

**DOI:** 10.2471/BLT.19.249656

**Published:** 2021-02-03

**Authors:** Shazia Moosa, Ammar Ali Muhammad, Sohail Dogar, Sundus Iftikhar, Walter Johnson, Asad Latif, Lubna Samad

**Affiliations:** a Center for Essential Surgical and Acute Care, Global Health Directorate, Indus Health Network, 5th Floor Woodcraft Building, Plot 3 & 3-A, Sector 47, Korangi Creek Road, Karachi, Pakistan.; b Department of Surgery, The Aga Khan University Hospital, Karachi, Pakistan.; c Indus Hospital Research Center, The Indus Hospital, Karachi, Pakistan.; d School of Public Health, Loma Linda University, Loma Linda, United States of America.; e Department of Anaesthesiology, The Aga Khan University Hospital, Karachi, Pakistan.

## Abstract

**Objective:**

To retrospectively review outcomes of a health provider-led infant circumcision programme in Pakistan.

**Methods:**

Based on World Health Organization guidelines, we trained surgical technicians and midwives to perform circumcisions using the Plastibell device at two Indus Health Network facilities. Programme tools include a training manual for health providers, information brochures for families, an enrolment form and standardized forms for documenting details of the procedure and outcomes. Infants aged 1–92 days were eligible for the study. Health workers contacted families on days 1 and 7 after the procedure to record any adverse events. We compared the characteristics of infants experiencing adverse events with infants facing no complications using multivariate logistic regression.

**Findings:**

Between August 2016 and August 2018, 2822 circumcised male infants with mean age 22.8 days were eligible for the study. Of these, 2617 infants (92.7%) were followed up by telephone interviews of caretakers. Older infants were more likely to experience adverse events than infants circumcised between 1–30 days of age: 31–60 days: adjusted odds ratio, aOR: 2.03; 95% confidence interval, CI: 1.31–3.15; 61–92 days: aOR: 2.14; 95% CI: 1.13–4.05. Minor adverse events (100 infants; 3.8%) included failure of the bell to shed (90 infants) and minimal bleeding (10 infants). Major adverse events (eight infants; 0.3%) included bleeding that required intervention (four infants), infection (three infants) and skin tear (one infant).

**Conclusion:**

Standardized training protocols and close monitoring enabled nonphysician health providers to perform safe circumcisions on infants aged three months or younger.

## Résumé

**Objectif:**

Procéder à un examen rétrospectif des résultats d'un programme de circoncision des nourrissons mené par des professionnels de santé au Pakistan.

**Méthodes:**

En nous fondant sur les directives de l'OMS, nous avons formé des techniciens en chirurgie et des sages-femmes afin qu'ils puissent opérer des circoncisions à l'aide du dispositif Plastibell dans deux établissements Indus Health Network. Les outils du programme comprennent un manuel de formation destiné aux professionnels de santé, des brochures d'information pour les familles, un formulaire d'inscription ainsi que des formulaires standardisés pour documenter les détails et résultats de la procédure. Seuls les nourrissons âgés de 1 à 92 jours ont été pris en compte dans cette étude. Les professionnels de santé ont contacté les familles le 1^er^ et le 7^e^ jour suivant la procédure afin de noter tout effet indésirable. Nous avons employé la régression logistique multivariée pour comparer les caractéristiques des nourrissons présentant des effets indésirables avec celles des nourrissons n'ayant subi aucune complication.

**Résultats:**

Entre août 2016 et août 2018, 2822 nourrissons circoncis âgés en moyenne de 22,8 jours répondaient aux conditions de participation à l'étude. Parmi eux, 2617 nourrissons (92,7%) ont fait l'objet d'un suivi par le biais d'entretiens téléphoniques de la part des soignants. Les nourrissons plus âgés étaient davantage susceptibles d'éprouver des effets indésirables que ceux circoncis entre 1 et 30 jours: 31–60 jours: odds ratio ajusté, ORA: 2,03; intervalle de confiance de 95%, IC: 1,31–3,15; 61–92 jours: ORA: 2,14; IC de 95%: 1,13-4,05. Les effets indésirables mineurs (100 nourrissons; 3,8%) incluaient l'échec de la séparation du gland (90 nourrissons) et des saignements limités (10 nourrissons). Les effets indésirables majeurs (huit nourrissons; 0,3%) incluaient des saignements nécessitant une intervention (quatre nourrissons), une infection (trois nourrissons) et une déchirure cutanée (un nourrisson).

**Conclusion:**

Des protocoles de formation standardisés ainsi qu'une surveillance étroite ont permis aux professionnels de santé autres que des médecins d'effectuer en toute sécurité des circoncisions sur des nourrissons âgés de maximum trois mois.

## Resumen

**Objetivo:**

Revisar retrospectivamente los resultados de un programa de circuncisión infantil temprana dirigido por proveedores sanitarios en Pakistán.

**Métodos:**

Según las directrices de la OMS, hemos formado a técnicos quirúrgicos y matronas para que realizaran circuncisiones con el dispositivo Plastibell en dos centros de la Red de Salud Indus. Las herramientas del programa incluyen un manual de formación para los proveedores sanitarios, folletos informativos para las familias, un formulario de inscripción y formularios estandarizados para documentar los detalles del procedimiento y los resultados. Los lactantes de 1 a 92 días de edad eran elegibles para el estudio. Los trabajadores sanitarios se pusieron en contacto con las familias los días 1 y 7 después del procedimiento para registrar cualquier acontecimiento adverso. Se compararon las características de los lactantes que experimentaron acontecimientos adversos con las de los lactantes que no sufrieron complicaciones mediante una regresión logística multivariante.

**Resultados:**

Entre agosto de 2016 y agosto de 2018, 2.822 bebés circuncidados con una media de edad de 22,8 días cumplieron los requisitos para participar en el estudio. De ellos, 2617 lactantes (92,7%) fueron objeto de seguimiento mediante entrevistas telefónicas por parte de los cuidadores. Los lactantes de más edad tuvieron más probabilidades de sufrir acontecimientos adversos que los circuncidados entre 1 y 30 días: 31-60 días: odds ratio ajustada, ORA: 2,03; intervalo de confianza del 95%, IC: 1,31-3,15; 61-92 días: ORA: 2,14; IC del 95%: 1,13-4,05. Los acontecimientos adversos menores (100 lactantes; 3,8%) incluyeron fallos en la separación del glande (90 lactantes) y hemorragias limitadas (10 lactantes). Los acontecimientos adversos importantes (ocho lactantes; 0,3%) incluyeron hemorragias que requirieron intervención (cuatro lactantes), infección (tres lactantes) y desgarro de la piel (un lactante).

**Conclusión:**

Los protocolos de formación estandarizados y la estrecha vigilancia han permitido a los profesionales sanitarios distintos de los médicos realizar con seguridad circuncisiones en bebés de hasta los tres meses de edad.

## ملخص

الغرض مراجعة نتائج برنامج الختان المبكر للرضع بأثر رجعي، تحت قيادة مقدم الرعاية الصحية في باكستان.

الطريقة بناءً على إرشادات منظمة الصحة العالمية (WHO)، قمنا بتدريب فنيي الجراحة والقابلات لإجراء عمليات الختان باستخدام جهاز Plastibell في اثنين من مرافق شبكة Indus الصحية. تتضمن أدوات البرنامج كتيبًا تدريبيًا لمقدمي الرعاية الصحية، ونشرات المعلومات للعائلات، واستمارة تسجيل، واستمارات قياسية لتوثيق تفاصيل ونتائج العملية. كان الرضع من عمر 1 إلى 92 يومًا، مؤهلين للدراسة. قام العاملون الصحيون بالاتصال بالأسر في اليومين الأول والسابع بعد العملية لتسجيل أي أحداث سلبية. قمنا بمقارنة خصائص الرضع الذين يمرون بالأحداث السلبية، مع الرضع الذين لا يواجهون أية مضاعفات باستخدام التحوف اللوجستي متعدد المتغيرات.

النتائج ما بين أغسطس/آب 2016 وأغسطس/آب 2018، كان 2822 من الرضع الذكور المختونين بمتوسط ​​عمر 22.8 يومًا، مؤهلين للدراسة. ومن بينهم، تمت متابعة 2617 رضيعًا (92.7%) عن طريق المقابلات الهاتفية مع القائمين على رعايتهم. كان الرضع الأكبر سنًا أكثر عرضة للتعرض لأحداث سلبية، من الأطفال المختونين بعمر 1 إلى 30 يومًا: 31 إلى 60 يومًا: نسبة الاحتمالات المعدلة، نسبة الأرجحية: 2.03؛ فاصل الثقة 95%: 1.31 إلى 3.15 يومًا؛ 61 إلى 92 يومًا: نسبة الاحتمالات المعدلة: 2.14؛ ‏بفاصل ثقة 95%: 1.13 إلى 4.05. تضمنت الأحداث السلبية الطفيفة (100 رضيع؛ 3.8%)، وشملت فشل الجرس في السقوط (90 رضيعًا)، ونزيفًا بسيطًا (10 أطفال). تضمنت الأحداث السلبية الرئيسية (ثمانية أطفال؛ 0.3%) النزيف الذي يتطلب تدخلاً (أربعة أطفال)، والعدوى (ثلاثة أطفال)، وتمزق الجلد (رضيع واحد).

الاستنتاج إن بروتوكولات التدريب القياسية والمراقبة عن كثب، أتاحت لمقدمي الرعاية الصحية من غير الأطباء، إجراء عمليات ختان آمنة للرضع من عمر ثلاثة أشهر أو أقل.

## 摘要

**目的:**

旨在回顾性地分析由医疗护理提供者主导的巴基斯坦早期婴儿包皮环切术计划的结果。

**方法:**

根据 WHO 的指导方针，我们在两个印度河卫生网络机构培训了一些外科技术人员和助产士，以确保其能够使用塑料钟环切器实施包皮环切术。该计划所采用的工具包括医疗护理提供者培训手册、家庭信息手册、登记表以及详细记录手术程序和手术结果的标准化表格。出生 1-92 天的婴儿符合本项研究的参与条件。医护工作者在术后第 1 天和第 7 天与家属联系，以记录任何不良事件。我们使用多元逻辑回归法比较了出现不良事件的婴儿与未出现并发症的婴儿的特征。

**结果:**

在 2016 年 8 月至 2018 年 8 月期间接受了包皮环切术的 2822 名平均年龄为 22.8 天的男婴符合本次研究的参与条件。看护人员对其中 2617 名婴儿 (92.7%) 进行了电话随访。与在出生 1-30 天内接受包皮环切术的婴儿相比，越大的婴儿越容易发生不良事件：31-60 天：调整后优势比，aOR：2.03；95% 置信区间，CI：1.31–3.15；61–92 天：aOR：2.14；95% CI：1.13-4.05。轻微不良事件（100 名婴儿；3.8%）包括塑料钟未脱落（90 名婴儿）和轻微出血（10 名婴儿）。严重不良事件（8 名婴儿；0.3%）包括需要干预的出血（4 名婴儿）、感染（3 名婴儿）和皮肤撕脱伤（1 名婴儿）。

**结论:**

标准化培训方案和严密监护使得并非医生的医疗护理提供者也能为 3 个月或更小的婴儿安全地实施包皮环切术。

## Резюме

**Цель:**

Выполнить ретроспективный обзор результатов программы раннего обрезания крайней плоти у младенцев мужского пола в Пакистане, проводимой провайдерами в сфере медико-санитарных услуг.

**Методы:**

Основываясь на рекомендациях ВОЗ, мы обучили фельдшеров хирургического отделения и акушерок выполнять обрезание крайней плоти с помощью устройства Plastibell в двух учреждениях сети Indus Health Network. Инструменты программы включают учебное пособие для провайдеров в сфере медико-санитарных услуг, информационные буклеты для семей, регистрационную форму и стандартные формы для документирования сведений о процедуре и результатах. Для исследования подходили младенцы в возрасте от 1 до 92 дней. Работники здравоохранения связывались с семьями на 1-й и 7-й дни после процедуры, чтобы зарегистрировать любые нежелательные явления. Мы сравнили характеристики младенцев, у которых возникли нежелательные явления, с младенцами, у которых не возникло осложнений, с использованием множественной логистической регрессии.

**Результаты:**

За период с августа 2016 года по август 2018 года 2822 обрезанных младенца мужского пола со средним возрастом 22,8 дня соответствовали критериям участия в исследовании. Из них для 2617 младенцев (92,7%) осуществлялся контроль в виде телефонных собеседований с опекунами. У младенцев старшего возраста вероятность возникновения нежелательных явлений выше, чем у младенцев, обрезанных в возрасте от 1 до 30 дней: в возрасте 31–60 дней скорректированное отношение шансов, сОШ: 2,03; 95%-й ДИ: 1,31–3,15; 61–92 дня: сОШ: 2,14; 95%-й ДИ: 1,13–4,05. Незначительные нежелательные явления (100 младенцев; 3,8%) включали неотделившуюся чашу устройства (90 младенцев) и минимальное кровотечение (10 младенцев). Основные нежелательные явления (восемь младенцев; 0,3%) включали кровотечение, которое потребовало вмешательства (четыре младенца), инфекцию (три младенца) и разрыв кожи (один младенец).

**Вывод:**

Стандартизованные протоколы обучения и тщательный мониторинг позволили провайдерам медико-санитарных услуг, которые не являются врачами, выполнять безопасное обрезание крайней плоти у младенцев в возрасте трех месяцев и младше.

## Introduction

Male circumcision is one of the most common surgical procedures performed, with an estimated 38.7% of males circumcised worldwide.^1^ Given an annual global birth rate of 70 million boys,^2^ around 27 million male circumcisions are performed per year, of which half are religiously or culturally motivated.^3^ In recent years, increasing evidence has linked male circumcision to lower rates of asymptomatic urinary tract infections, especially during infancy,^4^^,^^5^ and to a lower risk of sexually transmitted diseases, notably human immunodeficiency virus (HIV).^6^ This evidence has led to global attention on the prophylactic role of circumcision, with scale-up strategies being advocated in many countries with a high prevalence of HIV and acquired immunodeficiency syndrome,^7^ especially in sub-Saharan Africa. In Muslim-majority countries like Pakistan, male circumcision is considered an essential religious practice.^3^ This obligation contributes to large surgical volumes, making it even more important that circumcisions should be performed safely with the lowest possible risk of adverse events.

An estimated 3.2 million male infants are born in Pakistan every year,^8^ almost all of whom undergo circumcision in their infancy or childhood.^3^ The mean number of paediatric surgeons is 0.4 per million population in Pakistan^9^ and the number of paediatric surgeons in the country is about 200.^10^ Therefore, relying exclusively on specialists and general practitioners to meet the need for safe circumcisions is unfeasible. Currently, only 5–10% of boys are presented to qualified surgeons and physicians for circumcision.^11^ The remainder of caretakers approach traditional circumcisers, barbers and untrained paramedical staff,^12^^,^^13^ who often use unsterilized instruments and unsafe techniques with no follow-up or recording of adverse events.^11^

To promote safe circumcision, the World Health Organization (WHO) and the Johns Hopkins Program for International Education in Gynecology and Obstetrics developed a manual with technical guidance for structuring an infant circumcision programme. This manual recommends that infant male circumcision should be the primary task of nonphysician health-care workers.^14^ This is an example of task-sharing, where health providers, defined as nonphysician health-care workers by WHO, are trained to perform high volume, technically less demanding tasks, under close supervision and monitoring with a referral system in place.^14^^,^^15^ Such task-sharing between surgeons or physicians and trained health providers is not yet well-established in Pakistan, despite insufficient health-care resources. The country therefore needs a public health strategy in which appropriate nonphysician health providers are trained to perform circumcisions safely, using correct techniques and modern infection control practices.^16^

With these considerations in mind, the Safe Circumcision Programme was implemented at the Indus Hospital in Karachi to demonstrate a strategic approach for providing safe circumcisions to male infants in Pakistan. The programme provides training for surgical technicians and midwives to perform safe, sterile circumcisions following clearly defined programme guidelines based on recommendations from WHO^14^ and the American Academy of Pediatrics Task Force on Circumcision.^17^

This study aims to evaluate outcomes during the first 26 months of programme delivery.

## Methods

After a 4-month preparation phase (February through June 2016), the programme started in July 2016 at the Indus Health Network’s main campus, Indus Hospital, Karachi, Pakistan. In December 2017, the programme expanded to the network’s semi-urban facility in Muzaffargarh in Punjab Province, Pakistan. All Indus Health Network’s services, including circumcisions at the daily circumcision clinic, are provided free of charge. While the programme is ongoing, all procedures until 31 August 2018 were included in the present study. We obtained ethical approval for the study from Indus Hospital’s Institutional Review Board.

### Personnel and training

The programme has a coordinator responsible for the development of programme tools, training of health workers, data analysis and overall programme management. The training includes how to counsel caretakers, screen infants according to inclusion criteria and collect and enter data on paper forms and electronically using a computer software package. Paediatric surgeons train the health providers to perform circumcisions.

Programme tools include a training manual for health providers, information brochures for families, an enrolment form and standardized forms for documenting details of the procedure and outcomes. We developed visual aids, including pictorial representations of the procedure with possible adverse events and an animated video encouraging families to opt for early circumcisions. The video was played in the waiting rooms in the hospital to inform caretakers.

The training manual contains information about the programme’s background, related anatomy and surgical technique.^11^^,^^14^^,^^15^^,^^18^^–^^20^ The Plastibell method of circumcision is used since it is simple, easily taught, recommended by WHO^14^ and associated with few adverse events.^16^^,^^21^^–^^24^ Practical training started with observing circumcisions, followed by performing the procedure under supervision. We required each trainee to perform a minimum of 20 procedures independently to the trainer’s satisfaction. Upon completion of training, a paediatric surgeon assessed the trainee’s knowledge and skills. If the skills were suboptimal, the trainee needed to perform additional procedures until they reached a satisfactory level for certification.

Once certified, each health provider received a procedural fee of 200 Pakistani rupees (1.2 United States dollars, US$) per circumcision. For Karachi, we trained two surgical technicians, while for Muzaffargarh we initially trained three surgical technicians, who were later replaced by two trained midwives. 

### Patient selection

Caretakers of male infants born at facilities where the programme is established were counselled about early circumcision using programme brochures, while parents of infants born elsewhere spontaneously approached the facility for circumcision. Only healthy infants weighing at least 2.5 kg were included in the programme. 

Since circumcisions done in younger infants are simpler to perform, heal better, have fewer adverse events and provide health benefits earlier than when done in older infants,^25^^,^^26^ we originally set the upper age limit at 60 days, following WHO’s recommendations.^14^ Later, we extended this limit to 3 months, in agreement with the recommendation of Pakistani surgeons that circumcision should be performed within the first few months of life in the absence of contraindications.^13^ In underweight or unwell infants, we deferred circumcision until resolution of the underlying cause. We screened infants for genital anomalies, including hypospadias, epispadias, ambiguous genitalia, congenital buried penis, congenital chordee, micropenis or penoscrotal web, and asked questions for any signs or family history of bleeding disorders. We referred infants with an abnormality to a specialist. Preprocedure investigations, such as coagulation profile, are not routinely performed.^13^ We did not consider physiological jaundice as a contraindication; however, we deferred circumcision in infants with visually assessed deep jaundice.^12^^,^^27^

We avoided circumcision in the first 24 h after birth to ensure that the infant was stable, had time to void and had started to feed, as well as to give time for any abnormalities to become apparent.^14^

### Clinic routine

At the daily circumcision clinic, a health worker enrolled the infant, obtaining demographic information, medical history and informed consent from parents.^14^^,^^17^ The health worker recorded the infant’s vital signs. If documentation was not present regarding vitamin K administration at birth, the infant received one dose 30 minutes before the procedure.^28^^,^^29^

The health provider checked the equipment, verified the infant’s identity and performed a genital examination. A health worker either hand-restrained the infant or strapped him onto a circumcision board. The health provider administered a weight-adjusted dose of lidocaine for a dorsal penile nerve block before circumcision. Half an hour after the procedure, the health provider assessed the infant for excessive pain, swelling or bleeding. Parents received verbal information and a brochure, which included a helpline number, on postoperative care, possible causes for concern and the process for seeking urgent care.

### Outcome documentation

As part of the programme, a health worker called the caretaker on day 1 and day 7 after the procedure to enquire about the general well-being of the infant, ring shedding and parental satisfaction. If the ring had not shed by day 7, the health worker called on day 10 to instruct the caretaker to bring the infant to the hospital for ring removal. At least three calls and one text message, preferably to two different contact numbers, were sent before the infant was designated as lost to follow-up. Each health provider received feedback regarding recorded adverse events arising from their work. We classified adverse events as minor (easily handled by health providers); major (requiring specialist intervention); or serious (with life-threatening or long-term sequelae). To minimize bias, the health workers who collected outcome information differed from the health providers who performed the procedure. For monitoring and evaluation, the coordinator conducted periodic verification on randomly selected patients, by calling a few families every month to ask if they had received the follow-up calls and what their experience had been with the service being offered. Participants were de-identified by assigning programme identifiers, with personal data accessible to the programme team only.

### Data analysis

We entered all enrolment data in SPSS version 21.0 (IBM Corp., Armonk, New York, United States of America, USA). We expressed categorical variables as proportions, while quantitative variables were described by median and interquartile range, or mean and standard deviation (SD). To compare quantitative variables, we calculated Pearson’s correlation coefficients and for categorical variables we used *χ^2^* tests. The primary outcome variable was adverse events, scored as a binary response variable with yes = 1 and no = 0. We performed univariate and multivariate analyses, using logistic regression. All variables with *P* < 0.25 in univariate analyses were included in the final multivariable model. All analyses were performed with Stata version 14 (StataCorp LP, College Station, USA). A two-tailed *P* < 0.05 was considered significant.

## Results

**Fig. 1:**
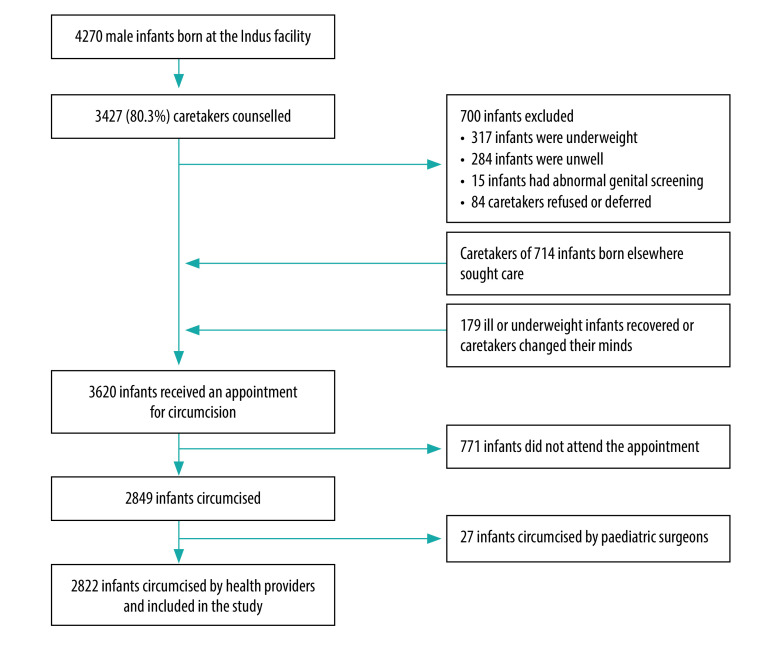
Flowchart for inclusion of male infants in the Indus Safe Circumcision Programme, Pakistan, 2016–2018

A total of 4270 boys were born at the two facilities during the study period and the caretakers of 80.3% (3427) of these were counselled about circumcision. During the screening process, 700 infants were excluded, of which 84 caretakers refused circumcision (Fig. 1). Reasons for refusal or deferral at the time of counselling included religious preference (i.e. non-Muslim parents), preference for local circumcisers, unwell mothers, mothers unable to decide independently or parents perceiving their infant too young for circumcision. Fifteen infants with genital abnormalities were referred to specialists. Fourteen infants with undescended testes, minimal chordee, minimal penile torsion, inguinal hernia or unilateral hydrocele were referred to a paediatric surgeon following circumcision. Of the 2822 infants circumcised, 2108 (74.7%) were born in a facility, whereas 714 were outpatients whose caretakers sought to circumcise their baby through the programme.

Table 1 shows demographic data of the 2822 enrolled infants. The age of circumcised infants ranged from 1 to 92 days (mean: 22.8; SD: 20.3) and their weight ranged from 2.5–6.7 kg (mean: 3.5; SD: 0.7). Most (2082 infants; 73.8%) were circumcised during the first month of life. Mean age at circumcision depended on the place of delivery and was 17.9 days (SD: 17.1) for inpatients, 36.9 days (SD: 22.1) for infants born at other health facilities, and 39.2 days (SD: 23.0) for infants delivered at home. Mild jaundice was noted in 320 infants (11.3%) at the time of procedure.

**Table 1:** Demographics of male infants in a task-sharing circumcision programme, Pakistan, 2016–2018

Characteristic	No. of infants (%) (*n* = 2822)
**Age at circumcision, days**	
1–30	2082 (73.8)
31–60	546 (19.3)
61–92	194 (6.9)
**Ethnicity**	
Sindhi	83 (2.9)
Mohajir	1401 (49.6)
Punjabi	379 (13.4)
Balochi	28 (1.0)
Pathan	122 (4.3)
Other	809 (28.7)
**Education status of father**	
No education	475 (16.8)
3–10 years of formal education	1527 (54.1)
> 10 years of formal education	820 (29.1)
**Education status of mother**	
No education	449 (15.9)
3–10 years of formal education	1592 (56.4)
> 10 years of formal education	781 (27.7)
**Religion of parents**	
Muslim	2712 (96.1)
Christian	109 (3.9)
Other	1 (< 0.1)
**Family structure**	
Joint family^a^	2035 (72.1)
Nuclear family	787 (27.9)
**Primary reason for circumcision**	
Religious	2716 (96.2)
Medical	88 (3.1)
Cultural/traditional	17 (0.6)
Other	1 (< 0.1)
**Place of delivery**	
The Indus Hospital	2108 (74.7)
Different facility	617 (21.9)
Home	97 (3.4)
**Presence of jaundice**	
Yes	320 (11.3)
No	2502 (88.7)

Note: Inconsistencies arise in some values due to rounding.

^a^ An extended family, typically consisting of three or more generations and their spouses, living together as a single household

Infants of Muslim caretakers comprised 96.1% (2712) of our study population, reflecting national statistics.^8^ Religious obligation was cited as a reason for undergoing circumcision by 96.2% (2716) of parents.

The most common Plastibell size used had a diameter of 1.2 cm (1076 infants; 38.1%), followed by 1.1 cm (750 infants; 26.6%) and 1.3 cm (587 infants; 20.8%). Telephone follow-up was possible for 2617 infants (92.7%). The ring shed spontaneously in 2520 infants (96.3%), with mean shedding day of 6.4 days (SD: 1.7). In 26 infants (1.0%) the ring hung by a small skin tag which shed spontaneously after day 10.

Following the procedure, 151 infants (5.4%) presented, spontaneously or after we telephoned the parents, to either the clinic or the emergency department. Of these, 43 caretakers required reassurance, mainly for concerns about ring impaction, bleeding, infection, swelling or unsatisfactory cosmesis. We documented adverse events in 108 (4.1%) infants of contactable caretakers, of which eight (0.3%) infants experienced major adverse events (Table 2). The most frequent adverse event was failure of the bell to shed (90 infants; 83.3%). Table 3 shows the comparison of different characteristics between infants experiencing an adverse event compared with infants who did not. After adjustment for other variables and compared with infants circumcised between 1–30 days after birth, infants circumcised when older were more likely to experience adverse events (31–60 days adjusted odds ratio, aOR: 2.03; 95% confidence interval, CI: 1.31–3.15; 61–92 days; aOR: 2.14; 95% CI: 1.13–4.05). Infants circumcised at the Muzaffargarh facility were more likely to experience adverse events than infants circumcised at the Karachi facility (aOR: 1.67, 95% CI: 1.09–2.54; Table 4).

**Table 2:** Reported adverse events after circumcision of male infants in a task-sharing circumcision programme, Pakistan, 2016–2018

Adverse event	No. of infants (%) (*n* = 2617)^a^
**All events**	108 (4.1)
**Major**	8 (0.3)
Bleeding requiring intervention^b^	4 (0.2)
Infection	3 (0.1)
Skin tear	1 (< 0.1)
**Minor**	100 (3.8)
Plastibell impaction	90 (3.4)
Bleeding settled with pressure and/or adrenaline	10 (0.4)

^a^ Sample size represents infants whose parents were reachable after the circumcision procedure.

^b^ Haemostasis was achieved by bipolar diathermy (one infant), placement of sutures (one infant), blood transfusion (one infant) or sutures plus blood transfusion (one infant).

**Table 3:** Comparison between circumcised male infants with or without an adverse event, in a task-sharing circumcision programme, Pakistan, 2016–2018

Characteristic	No. of infants with adverse event (%) (*n* = 108)	No. of infants without adverse event (%) (*n* = 2714)	*P*^a^
**Age at circumcision, days**			
1–30 (*n* = 2082)	63 (3.0)	2019 (97.0)	0.002
31–60 (*n* = 546)	32 (5.9)	514 (94.1)	
61–92 (*n* = 194)	13 (6.7)	181 (93.3)	
**Presence of jaundice**			
No (*n* = 2502)	102 (4.1)	2400 (95.9)	0.057
Yes (*n* = 320)	6 (1.9)	314 (98.1)	
**Ethnicity**			
Muhajir (*n* = 1401)	56 (4.0)	1345 (96.0)	0.317
Balochi (*n* = 28)	1 (3.6)	27 (96.4)	
Pathan (*n* = 122)	3 (2.5)	119 (97.5)	
Punjabi (*n* = 379)	7 (1.8)	372 (98.2)	
Sindhi (*n* = 83)	4 (4.8)	79 (95.2)	
Other (*n* = 809)	37 (4.6)	772 (95.4)	
**Place of delivery**			
The Indus Hospital (*n* = 2108)	73 (3.5)	2035 (96.5)	0.213
Different facility (*n* = 617)	28 (4.5)	589 (95.5)	
Home (*n* = 97)	7 (7.2)	90 (92.8)	
**Father's education**			
No education (*n* = 475)	18 (3.8)	457 (96.2)	0.979
3–10 years of education (*n* = 1527)	58 (3.8)	1469 (96.2)	
> 10 years of education (*n* = 820)	32 (3.9)	788 (96.1)	
**Mother's education**			
No education (*n* = 449)	22 (4.9)	427 (95.1)	0.562
3–10 years of education (*n* = 1592)	58 (3.6)	1534 (96.4)	
> 10 years of education (*n* = 781)	28 (3.6)	753 (96.4)	
**Site of programme**			
Karachi (*n* = 2203)	75 (3.4)	2128 (96.6)	0.023
Muzaffargarh (*n* = 619)	33 (5.3)	586 (94.7)	

^a^ One-tailed χ^2^ test (univariate analysis).

**Table 4:** Factors associated with an adverse event following infant male circumcision in a task-sharing circumcision programme, Pakistan, 2016–2018

Independent variable^a^	Unadjusted OR (95% CI)^b^	Adjusted OR (95% CI)^c^
**Age at circumcision, days**		
1–30	Ref.	Ref.
31–60	1.99 (1.28–3.08)	2.03 (1.31–3.15)
61–92	2.11 (1.11–3.99)	2.14 (1.13–4.05)
**Presence of jaundice**		
No	Ref.	Ref.
Yes	0.45 (0.19–1.04)	0.66 (0.27–1.56)
**Place of delivery**		
The Indus Hospital	Ref.	Ref.
Different facility	1.33 (0.85–2.07)	1.13 (0.69–1.85)
Home	1.84 (0.78–4.34)	1.38 (0.57–3.34)
**Site of programme**		
Karachi	Ref.	Ref.
Muzaffargarh	1.62 (1.06–2.46)	1.67 (1.09–2.54)

CI: confidence interval; OR: odds ratio; Ref: reference.

^a^ We included variables with a *P*-value less than 0.25 in Table 3.

^b^ Univariate binary logistic regression.

^c^ Multivariable binary logistic regression (adjusting for all other exploratory variables).

Note: Outcome variable was occurrence of adverse events: yes = 1, no = 0.

The rate of parental satisfaction with the programme was 99.8% (2611/2617).

## Discussion

While some believe that the Plastibell circumcision method is only safe when performed by experienced surgeons,^30^ task-sharing is needed to provide safe circumcision for infants in countries with large volumes due to high birth rate and increased demands mainly due to religious considerations. To meet the demand in Pakistan, we used the WHO task-sharing recommendations for infant male circumcision services^14^ to train nonphysician health providers to circumcise boys aged 3 months or younger. Such task-sharing has also been successfully implemented in African countries where nurses and trained paramedical staff routinely perform adult male circumcisions^31^^–^^33^ and in Bradford, the United Kingdom of Great Britain and Northern Ireland, to cater to the Muslim population.^19^^,^^34^

Our study shows an overall proportion of adverse events of 4.1%, of which only 0.3% required specialist intervention and none were life-threatening. This proportion is lower than reported proportions for similar studies in other settings. In two studies based in the United Kingdom, infants aged 6 to 14 weeks underwent Plastibell circumcisions by trained nurses and the reported proportions of adverse events were 8.2% (93/1129) and 18.5% (31/168).^19^^,^^34^ In a large-scale study in Nigeria, where trained doctors employed the Plastibell method of circumcision in neonates and infants, the proportion was 1.1% (25/2276).^22^ In contrast, a single-surgeon series of 381 infants undergoing Plastibell circumcisions in the Islamic Republic of Iran showed a proportion of adverse events of 7.1% (27/381),^35^ whereas 386 infants circumcised by the same method by a paediatrician in the United Republic of Tanzania showed only minor adverse events in 2.8% (11/386) of infants.^24^

Postoperative adverse events seem to be independent of technique, whether procedures are performed in high- or low-income settings, or if done by a surgeon, paediatrician, nurse or health worker.^17^ Thorough training of the health provider, meticulous technique when performing the circumcision and close monitoring of outcomes to ensure consistent quality of care appear to be key for preventing adverse events.^11^^,^^18^^–^^20^

The risk of adverse events using the Plastibell method increases with age of the infant,^21^^,^^36^ which can be attributed to increasing thickness and vascularity of preputial skin and difficulty in restraining larger infants.^24^ Our results indicate that infants circumcised within their first month of life experienced the least number of adverse events. Although higher, the proportion of adverse events remained constant in infants after the neonatal period till 3 months of age. By extending the age limit to 3 months in our programme, we were able to perform safe circumcisions for infants who had surpassed the 60 days age limit set by WHO.^14^ The mean age at circumcision was lower for inpatient infants, indicating that partnering circumcision clinics with maternity services or vaccination centres facilitates counselling and earlier referral.

Adverse events arising after Plastibell circumcision range from trivial to consequential.^20^ The most common adverse event in our study was failure of the ring to shed or ring impaction, similar to other studies (3.6%; 41/1129 and 6.1%; 75/1223 respectively).^19^^,^^37^ A health worker addressed this adverse event with a quick, atraumatic intervention, cutting the ring without anaesthesia. Selection of bell size depended on visual estimation of the width of the glans, which is expected to become more accurate with experience. No infant in our study experienced serious ring-related adverse events, although such events have been reported by others.^38^^–^^41^ Post-procedure bleeding was reported in 1.1% (30/2625) of children in a large retrospective review;^42^ our study showed an incidence of 0.5% which also included minor bleeds that were controlled by application of direct pressure with gauze or gauze sprayed with adrenaline. While oral antibiotics are prescribed routinely in some centres,^22^ the nominal infection rate observed in our programme justifies our practice of avoiding the prophylactic use of postoperative oral antibiotics.

The significant difference observed in proportions of adverse events between the Karachi and Muzaffargarh sites may be attributed to the fact that the programme team remotely monitored this site, while the Karachi site received close support, as the programme team was based in Karachi. Additionally, unlike the Muzaffargarh site, on-campus paediatric surgeons were available in Karachi to intervene early in complicated cases.

In our opinion, factors critical to the programme’s success are thorough training of health providers, close monitoring of outcomes and a reliable referral system. Additionally, strict adherence to programme protocols, which was ensured by monitoring, and provision of clear follow-up instructions to families provided a safety net for patient care. Key challenges during implementation were to maintain an uninterrupted supply of Plastibells, continuous availability of sterilized instruments and to obtain permission from provincial governments to allow nonphysicians to perform the procedure. These challenges were addressed by advance planning, volume projections, close coordination with the supply chain and the Central Sterile Supply Department. Furthermore, to convince the government and regulatory bodies to allow task-sharing, we provided them with evidence-based results, including programme data. 

Notably, this programme could have potential cost savings for the health system and families. Cost of circumcision done by paediatric surgeons at local private hospitals ranges from 3600 to 9000 Pakistani rupees (US$ 22.4–55.9) per procedure, whereas public sector hospitals provide the same service free of cost, albeit with long waiting lists and overburdened teams. In comparison, one procedure in our programme costs 1550 Pakistani rupees (US$ 9.6), which includes the procedural fee plus cost of supplies, consumables and indirect cost. If scaled up to national level, our approach can ensure safe circumcisions at lower costs to both the health system and families.

Between March and June 2020, health-care resources at the network’s facilities were redirected to treat patients with coronavirus disease 2019 and all elective surgeries were suspended. This suspension created significant surgical backlog including circumcisions. When routine surgeries gradually resumed, the paediatric surgery team had to prioritize essential surgeries over circumcisions of infants who had surpassed the age limit of inclusion in our programme during the pause in routine services. We overcame this challenge by increasing the age limit to 6 months and included this cohort of infants in the Safe Circumcision Programme, while supported by the paediatric surgery team in case of any adverse event. This experience provides an excellent example of how task-sharing can fill a gap in the delivery of a key service at a time of critical need.

This study had some limitations. We derived the data from a service delivery programme, which adopted a single method of circumcision most suited for our population. Outcome data are missing for 7% (205) of infants because their parents could not be contacted after the procedure. Adverse events may have been overreported in our study, since we counted all instances of bleeding, including minimal ooze, unlike previous studies. Since shedding of the ring was the end-point for follow-up, long-term sequelae are not reported here.

We have shown that infant circumcisions are safely performed within 3 months of birth by trained health providers. In high-volume settings with insufficient numbers of surgeons, trained surgical technicians and midwives are a reliable resource for performing circumcisions if standardized training protocols and close monitoring systems are in place.
